# Sexual dysfunction among early-onset colorectal cancer survivors: Sex-specific correlates of sexual health discussions between patients and providers

**DOI:** 10.1007/s10552-023-01772-1

**Published:** 2023-08-19

**Authors:** Julia Stal, Serena Y. Yi, Sally Cohen-Cutler, Phuong Gallagher, Afsaneh Barzi, David R. Freyer, Jonathan N. Kaslander, Martina Anto-Ocrah, Heinz-Josef Lenz, Kimberly A. Miller

**Affiliations:** 1 Department of Population and Public Health Sciences, Keck School of Medicine, University of Southern California, 1845 N. Soto Street, 3rd Floor, Los Angeles, CA 90032 USA; 2 Cancer and Blood Disease Institute, Children’s Hospital Los Angeles, Los Angeles, CA USA; 3 Department of Pediatrics, Keck School of Medicine, University of Southern California, Los Angeles, CA USA; 4 The Colon Club, Pasadena, CA USA; 5 Department of Medical Oncology and Therapeutics Research, City of Hope National Medical Center, Duarte, CA USA; 6 USC Norris Comprehensive Cancer Center, Los Angeles, CA USA; 7 Division of General Internal Medicine, Department of Medicine, University of Pittsburgh, Pittsburgh, PA USA; 8 Division of Oncology, University of Southern California Norris Comprehensive Cancer Center, Los Angeles, CA USA; 9 Department of Dermatology, Keck School of Medicine, University of Southern California, Los Angeles, CA USA

**Keywords:** Sexual health, Sexual dysfunction, Erectile dysfunction, Colorectal cancer, Cancer survivorship, Young adults

## Abstract

**Purpose:**

To examine the prevalence of female sexual dysfunction (FSD), male erectile dysfunction (ED), and the prevalence and correlates of sexual health discussions between early-onset CRC survivors and their health care providers.

**Methods:**

An online, cross-sectional survey was administered in partnership with a national CRC advocacy organization. Respondents (n = 234; diagnosed < 50 years, 6–36 months from diagnosis/relapse) were colon (36.8%) and rectal (63.3%) cancer survivors (62.5% male). The Female Sexual Function Index (FSFI-6) was used to measure FSD, and the International Index of Erectile Function (IIEF-5) was used to measure ED. Survivors reported whether a doctor communicated with them about sexual issues during/after treatment.

**Results:**

Among females (n = 87), 81.6% had FSD (mean FSFI-6 score = 14.3 [SD±6.1]). Among males (n = 145), 94.5% had ED (mean IIEF-5 score = 13.6 [SD±3.4]). Overall, 59.4% of males and 45.4% of females reported a sexual health discussion. Among the total sample, older age of diagnosis and relapse were significantly associated with reporting a discussion, while female sex was negatively associated with reporting a sexual health discussion. Among males, older age at diagnosis and relapse, and among females, older age of diagnosis, were significantly associated with reporting a sexual health discussion.

**Conclusion:**

The prevalence of FSD and ED were high (8 in 10 females reporting FSD, almost all males reporting ED), while reported rates of sexual health discussion were suboptimal (half reported discussion). Interventions to increase CRC provider awareness of patients at risk for not being counseled are needed to optimize long-term health outcomes.

## Background

The incidence of early-onset (diagnosed under 50 years) CRC has increased in the United States annually by 1.27% from 2001 to 2012 and by 3.00% from 2012 to 2017 [1]. Among this population, this incidence has increased for tumors in the proximal and distal colon and rectum and is driven by non-Hispanic white patients [2]. Fortunately, nearly 7 in 10 early-onset CRC patients will survive at least 5 years post-diagnosis [2]. However, intensive, multimodal cancer therapies, including aggressive surgery, chemotherapy and/or radiation, can negatively affect survivors’ sexual health, sexual response (i.e., desire, arousal, climax, and resolution), and impair quality of life [3–5].

Sexual health-related late effects among cancer survivors include physical domains such as erectile dysfunction (among males; ED), the most common form of male sexual dysfunction, vaginal dryness (among females), or an inability to reach orgasm (among both males and females), as well as psychological domains such as poorer mental health and high levels of distress [6]. Fortunately, sexual health-related late effects can be reduced or mitigated with proper counseling and intervention before, during, and after treatment [6]. Although the prevalence of sexual dysfunction among adult-onset cancer survivors has been well-described, studies have seldom focused on early-onset CRC patients who have a greater risk of sexual dysfunction due to both their life stage (i.e., age and maturity) and cancer experience (i.e., a primary tumor in the abdominopelvic region treated with intensive therapy that may damage nerve function and/or affect hormone levels) [7, 8]. Consequently, sexual health is a particular concern for early-onset CRC patients as they begin to explore romantic relationships, discuss sexual health with partners, and navigate treatment-related sexual dysfunction [9, 10].

Both the National Comprehensive Cancer Network (NCCN) and the American Society of Clinical Oncology (ASCO) clinical guidelines indicate that providers should discuss sexual function with patients at regular intervals [11, 12]. Specifically, ASCO indicates sexual health should be discussed at time of diagnosis and reassessed periodically throughout follow-up care [12]. Despite clinical guidelines, only 4 in 10 adult CRC survivors report receiving information on how cancer or its treatment may affect their sex life, with females less likely to receive sexual health information than males, suggesting survivors experience barriers to obtaining proper sexual health counseling [13]. Often, patients and providers alike are unsure who should initiate conversations about sexual health [14]. Provider-level barriers to discussing sexual health within the oncology setting include a lack of time, knowledge, and/or training [15]. Patient-level barriers to discussing sexual health include wanting providers to initiate conversation [16], feeling there are more important issues to discuss with their oncologist [16], and a perceived lack of respect from their provider [17]. However, much of the existing literature focuses on survivors of adult-onset cancer or survivors of a variety of cancer types. As such, we lack an understanding of the nuances of sexual dysfunction and sexual health discussions as they pertain directly to early-onset CRC survivors.

The present study sought to examine the prevalence of FSD, ED, and the prevalence and correlates of sexual health discussions between early-onset male and female CRC survivors under age 50 and their providers. Consistent with prior research among adult-onset CRC survivors [13, 18], we hypothesized there would be high rates of sexual dysfunction among both early-onset male and female CRC survivors and low rates of sexual health discussions between survivors and their providers. Further, we hypothesized that male sex [19, 20] and greater treatment intensity [21] would be associated with reporting a sexual health discussion with a provider.

## Methods

An online, cross-sectional survey was administered on the Facebook page of a national CRC advocacy organization, *The Colon Club*, using Research Electronic Data Capture (REDCap) between August 31st and September 3rd, 2020 [22–24]. The Facebook page contains roughly 7,000 members and is in English only; as such, the survey was only available in English. Respondents were asked a series of questions to determine their eligibility, and if eligible (colon or rectal cancer survivor, early-onset [under age 50] at diagnosis, 6 to 36 months from diagnosis or relapse, based in the United States), were asked to provide consent to participate. Participants received a $20 electronic gift card upon survey completion. Steps to ensure data validity and integrity and to prevent fraudulent responses were utilized, such as prohibiting the use of duplicate email addresses, removing responses with a survey completion time substantially below the mean of 17 min (< 5 min), and removing responses determined by a medical oncologist (A.B.) to have improbable cancer treatment patterns [25]. Additional steps and study procedures are detailed elsewhere [22–24]. The study was approved by the University of Southern California (USC) Institutional Review Board (IRB).

### Measures and Correlates

Demographics: Respondents reported current age, age at diagnosis, gender (*woman, man, transgender*, or *a gender not listed/other* [those who identified as transgender or other were able to select a male- or female-specific survey], race/ethnicity (*white, Hispanic/Latino/ Latinx, Black or African American, Asian, Native Hawaiian or Pacific Islander, American Indian* or *Alaska Native*), marital status (*single, living with a partner, married, widowed, or divorced/separated*), employment status (*full-time, part-time, stay at home parent, student, unemployed or disabled, or other*), and highest level of education (*some high school or less, high school graduate or GED, some college training or Associates Degree, college graduate*, or *post-graduate training*).

Clinical Factors: Respondents reported cancer type (*colon/rectal*), stage at diagnosis (*1 to 4*), treatment type (*chemotherapy, radiation, surgery, and/or immunotherapy* [participants were able to endorse more than one treatment type]), if their cancer relapsed, and if they have an ostomy (*yes/no*).

Female Sexual Dysfunction: The Female Sexual Function Index (FSFI-6) was used to screen for FSD among females [26]. Female participants responded to six sexual function items pertaining to the past four weeks utilizing a Likert-type scale, with response options specific to each item [26]. The FSFI-6 was found to have good internal consistency in prior research (α = 0.79) [26] and excellent internal consistency in the present study (α = 0.90). FSFI-6 scores range from 2 to 30, with lower scores indicating worse sexual function [26]. A score of 19 or less was found to have good discrimination (sensitivity = 0.93, specificity = 0.94) and was used to indicate FSD in the present study [26].

Male Erectile Dysfunction: The International Index of Erectile Function (IIEF-5) was used to screen for ED among males [27, 28]. Male participants responded to five ED items pertaining to the past four weeks utilizing a Likert-type scale, with varying response options specific to each item [27]. The IIEF-5 was found to have excellent internal consistency both in prior research (α = 0.90) [28] and in the present study (α = 0.81). IIEF-5 scores range from 5 to 25 and, in accordance with prior research, the following classifications were used in the present study: (5 to 7), moderate (8 to 11), mild to moderate (12 to 16), mild (17 to 21), and no ED (22 to 25) [27]. An IIEF-5 score of 21 or less was found to have good discrimination (sensitivity = 0.98, specificity = 0.88) and was used to indicate ED in the present study [27].

Sexual Health Discussions: Both male and female survivors were asked to indicate, “Has a doctor ever talked to you about sexual issues during or after treatment” (*yes, no, not sure*).

### Statistical Analysis

Race/ethnicity was dichotomized to represent non-Hispanic/Latino white and survivors of color due to small numbers of the latter. Employment was dichotomized to represent working full-time versus working part-time or less. Education was dichotomized to represent a high school graduate or less versus some college or more. Treatment intensity was analyzed as the summed number of treatment modalities endorsed by each respondent (ranging from 0 to 4).

Descriptive statistics were utilized to examine sample demographics and frequencies of item responses. Logistic regression was used to identify factors associated with having a sexual health discussion and to generate effect estimates. Variables (i.e., sociodemographic, clinical factors) were selected for their hypothesized significance to the outcome and were used for bivariate analysis. Variables significant at *p* < .10 were included in the multivariable logistic regression model. Tests were two-tailed, with an alpha criterion of *p* < .05. Analysis was performed using Stata (Version 15.1, StataCorp, College Station, Texas).

## Results

A total of 234 early-onset colon or rectal cancer survivors were included in the present study (Table 1). Survivors had a mean current age of 34.6 years (SD±6.6; range 20 to 49) and a mean age at diagnosis of 32.7 years (SD±6.7; range 17 to 48). Survivors were primarily diagnosed with rectal cancer (63.3%), stage 2 (59.5%), and were primarily treated with radiation (56.8%).

**Table 1:** Sample characteristics*

	Male (N = 145)	Female (N = 87)	Total (N = 234)
	N (%) or M (SD)^	N (%) or M (SD)	N (%) or M (SD)
**Sociodemographic Factors**			
**Current Age (M[SD])** ^**a**^	34.8 (6.9)	34.5 (6.1)	34.6 (6.6)
**Age of Diagnosis (M[SD])** ^**a**^	33.0 (7.0)	32.5 (6.1)	32.7 (6.7)
**Race/Ethnicity** ^**b**^			
White	113 (77.9)	67 (78.8)	180 (77.9)
Black or African American	12 (8.3)	11 (12.9)	23 (10.0)
Hispanic/ Latino/ Latinx	16 (11.0)	6 (7.1)	22 (9.5)
Asian	2 (1.4)	.	2 (0.9)
Native Hawaiian or Pacific Islander	1 (0.7)	1 (1.2)	3 (1.3)
American Indian or Alaska Native	1 (0.7)	.	1 (0.4)
**Marital Status** ^**b**^			
Single (never married)	22 (15.2)	18 (20.7)	41 (17.5)
Living with a partner	27 (18.6)	14 (16.1)	41 (17.5)
Married	95 (65.5)	53 (60.9)	149 (63.7)
Widowed	1 (0.7)	.	1 (0.4)
Divorced/separated	.	2 (2.3)	2 (0.9)
**Employment** ^**b**^			
Working full-time	82 (56.6)	37 (42.5)	119 (50.9)
Working part-time	52 (35.9)	39 (44.8)	92 (39.3)
Stay-at-home parent	2 (1.4)	3 (3.5)	6 (2.6)
Unemployed or permanently disabled	7 (4.8)	5 (5.8)	12 (5.1)
Other	2 (1.4)	3 (3.5)	5 (2.1)
**Highest Level of Education** ^**b**^			
Some high school or less (< 12 years)	10 (6.9)	7 (8.1)	18 (7.7)
High school graduate or GED (12 years)	13 (9.0)	7 (8.1)	21 (9.0)
Some college training or Associates Degree	95 (66.0)	63 (72.4)	158 (67.8)
College graduate or more**	26 (18.1)	10 (11.5)	36 (15.5)
**Clinical Factors**			
**Cancer Type** ^**b**^			
Colon	49 (33.8)	36 (41.4)	86 (36.8)
Rectal	96 (66.2)	51 (58.6)	148 (63.3)
Treatment*** ^**b**^			
Chemotherapy	51 (35.2)	31 (35.6)	82 (35.0)
Radiation	82 (56.6)	51 (58.6)	133 (56.8)
Surgery	75 (51.7)	47 (54.0)	124 (53.0)
Immunotherapy	44 (30.3)	20 (23.0)	64 (27.4)
**Treatment Intensity (M[SD])** ^**a**^	1.74 (0.9)	1.7 (0.8)	1.7 (0.9)
**Relapsed** ^**b**^	89 (61.4)	53 (61.6)	143 (61.4)
**Stage of Diagnosis** ^**b**^			
Stage 1	22 (15.3)	20 (23.3)	43 (18.5)
Stage 2	91 (63.2)	47 (54.7)	138 (59.5)
Stage 3	28 (19.4)	16 (18.6)	45 (19.4)
Stage 4	3 (2.1)	3 (3.5)	6 (2.6)
**Have Ostomy** ^**b**^	53 (37.1)	28 (32.9)	82 (35.7)
**Sexual Health Factors**			
**Discussed sexual health with their provider** ^**b**^	85 (59.4)	39 (45.4)	124 (54.2)
**Female Sexual Function Index (FSFI-6) Score (M[SD])** ^**a**^	.	14.3 (6.1)	.
**International Index of Erectile Function (IIEF-5) Score (M[SD])** ^**a**^	13.6 (3.4)	.	.

^a^Denotes continuous variable; ^b^Denotes categorical variable

^M(SD) represents mean and standard deviation

*Total values may not sum to N = 234 due to item missingness; **Includes BA/BS, MA/MS, PhD, MD, or other graduate degree; ***Some respondents endorsed more than one treatment type

### Sexual Dysfunction

**Females**: Among female early-onset CRC survivors (n = 87), eight in ten (81.6%) had FSD. Table 2 provides frequencies for FSFI-6 item responses. Females had a mean FSFI-6 score of 14.3 (SD±6.1). The majority of female survivors endorsed the following over the past four weeks: *moderate* (40.0%) sexual desire or interest (41.2% reported *very low or none at all* or *low* desire or interest, cumulatively), *moderate* (34.5%) sexual arousal (‘turn on’) during sexual activity or intercourse (42.5% reported *very low or none at all* or *low* sexual arousal, cumulatively), became lubricated (“wet”) during sexual activity or intercourse *a few times* or *sometimes*, equally (29.1% each; 12.8% reported *almost never or never* becoming lubricated), reaching orgasm *a few times* (33.3%) during sexual stimulation or intercourse (11.5% reported *almost never or never* reaching orgasm), are *about equally satisfied* (neutral; 31.0%) with their overall sexual life (42.5% reported being *very dissatisfied* or *moderately dissatisfied* with their sexual life, cumulatively), and experiencing discomfort or pain during vaginal penetration *most times* (32.2%; 14.9% reported *almost always or always* experiencing discomfort or pain). 

**Table 2:** Female sexual function index (FSFI-6)

	N (%)	N (%)	N (%)	N (%)	N (%)	N (%)
	No sexual activity	Very low or none at all	Low	Moderate	High	Very high
**How would you rate your level (degree) of sexual desire or interest?**	.	14 (16.47)	21 (24.71)	34 (40.0)	16 (18.82)	0
**How would you rate your level of sexual arousal (“turn on”) during sexual activity or intercourse?**	13 (14.94)	9 (10.34)	28 (32.18)	30 (34.48)	7 (8.05)	0
	**No sexual activity**	**Almost never or never**	**A few times**	**Sometimes**	**Most times**	**Almost always or always**
**How often did you become lubricated (“wet”) during sexual activity or intercourse?**	16 (18.60)	11 (12.79)	25 (29.07)	25 (29.07)	9 (10.47)	0
**When you had sexual stimulation or intercourse, how often did you reach orgasm?**	16 (18.39)	10 (11.49)	29 (33.33)	25 (28.74)	7 (8.05)	0
	.	**Very dissatisfied**	**Moderately dissatisfied**	**About equally satisfied**	**Moderately satisfied**	**Very satisfied**
**How satisfied have you been with your overall sexual life?**	.	16 (18.39)	21 (24.14)	27 (31.03)	22 (25.29)	1 (1.15)
	**Did not attempt intercourse**	**Almost never or never**	**A few times**	**Sometimes**	**Most times**	**Almost always or always**
**How often did you experience discomfort or pain during vaginal penetration?**	17 (19.54)	1 (1.15)	3 (3.45)	25 (28.74)	28 (32.18)	13 (14.94)

**Males**: Among male early-onset CRC survivors (n = 145), 94.5% had ED. Table 3 provides frequencies for IIEF-5 item responses. Males had a mean IIEF-5 score of 13.6 (SD±3.4). The majority of male survivors endorsed the following over the past four weeks: *moderate* (45.1%) confidence that they could get and keep an erection (33.1% reported *very low* or *low* confidence, cumulatively), erections hard enough for penetration *almost never/never* or *a few times (much less than half the time;* 37.9%, cumulatively), ability to maintain erection after they had penetrated (entered) their partner *almost never/never* or *a few times (much less than half the time;* 40.1%, cumulatively), had satisfactory intercourse *almost never/never* or *a few times (much less than half the time;* 34.3%, cumulatively), and *difficulty* (33.3%) maintaining an erection to completion of intercourse (37.5% reported this to be *verydifficult* or *extremely difficult*, cumulatively).  

**Table 3:** International index of erectile function (IIEF-5)

	N (%)	N (%)	N (%)	N (%)	N (%)
	**Very low**	**Low**	**Moderate**	**High**	**Very high**
**How do you rate your confidence that you could get and keep an erection?**	12 (8.45)	35 (24.65)	64 (45.07)	27 (19.01)	4 (2.82)
	**Almost never / never**	**A few times (much less than half the time)**	**Sometimes (about half the time)**	**Most times (much more than half the time)**	**Almost always / always**
**When you had erections with sexual stimulation, how often were your erections hard enough for penetration?**	15 (10.34)	37 (25.52)	82 (56.55)	11 (7.59)	0
**During sexual intercourse, how often were you able to maintain your erection after you had penetrated (entered) your partner?**	20 (13.89)	39 (27.08)	68 (47.22)	15 (10.42)	2 (1.39)
**When you attempted sexual intercourse, how often was it satisfactory for you?**	18 (12.59)	31 (21.68)	78 (54.55)	14 (9.79)	2 (1.40)
	**Extremely difficult**	**Very difficult**	**Difficult**	**Slightly difficult**	**Not difficult**
**During sexual intercourse, how difficult was it to maintain your erection to completion of intercourse?**	10 (6.94)	44 (30.56)	48 (33.33)	38 (26.39)	4 (2.78)

### Sexual Health Discussions and Correlates

Overall, 54.2% of early-onset CRC survivors reported that their doctor had talked to them about sexual issues during or after treatment (45.4% females, 59.4% males). Table 4 provides bivariate and multivariable models of correlates of sexual health discussions among young adult CRC survivors and their providers.

**Table 4:** Sex-specific bivariate and multivariable models of correlates of sexual health discussions between early-onset colorectal cancer survivors and their providers

	Males (N = 145)		Females (N = 87)		Total (N = 234)*	
	OR [95% CI]	AOR [95% CI]	OR [95% CI]	AOR [95% CI]	OR [95% CI]	AOR [95% CI]
**Sociodemographic Factors**						
**Sex**						
Female	.	.	.	.	0.57 [0.33, 0.97]*	0.52 [0.29, 0.95]*
Male	.	.	.	.	1.0	1.0
**Age of Diagnosis**	1.07 [1.02, 1.13]**	1.07 [1.01, 1.13]*	1.14 [1.05, 1.24]**	1.14 [1.03, 1.26]**	1.09 [1.05, 1.12]***	1.06 [1.01, 1.11]*
**Race/Ethnicity**						
White	0.71 [0.31, 1.62]	.	0.37 [0.12, 1.12]+	0.52 [0.15, 1.78]	0.56 [0.29, 1.07]+	0.74 [0.36, 1.52]
Respondent of color^	1.0	.	1.0	1.0	1.0	1.0
**Marital Status**						
Partner	1.96 [0.78, 4.89]	.	0.78 [0.29, 2.14]	.	1.37 [0.71, 2.70]	.
No partner	1.0	.	1.0	.	1.0	.
**Level of Education**						
High school graduate or less	0.56 [0.23, 1.37]	.	0.42 [0.12, 1.47]	.	0.51 [0.25, 1.05]+	0.53 [0.24, 1.12]
Some college or more	1.0	.	1.0	.	1.0	1.0
**Clinical Factors**						
**Type of Cancer**						
Colon	1.91 [0.92, 3.97]+	1.97 [0.88, 4.44]+	0.94 [0.40, 2.22]	.	1.35 [0.79, 2.32]	.
Rectal	1.0	1.0	1.0	.	1.0	.
**Treatment Intensity**	1.00 [0.68, 1.46]	.	0.68 [0.39, 1.18]	.	0.89 [0.65, 1.20]	.
**Stage Diagnosed (1–4)**	0.75 [0.45, 1.26]	.	1.25 [0.70, 2.24]	.	0.96 [0.65, 1.40]	.
**Relapse**						
Yes	3.78 [1.86, 7.70]***	3.08 [1.39, 6.81]**	1.73 [0.71, 4.24]	.	2.75 [1.59, 4.77]***	2.28 [1.20, 4.34]*
No	1.0	1.0	1.0	.	1.0	1.0
**Ostomy**						
Yes	2.31 [1.12, 4.98]*	1.18 [0.51, 2.73]	2.58 [1.02, 6.54]*	0.84 [0.26, 2.71]	2.43 [1.37, 4.30]**	1.14 [0.58, 2.22]
No	1.0	1.0	1.0	1.0	1.0	1.0

+P < .10; *P < .05, **P < .01, ***P < .001

*Total values may not sum to N = 234 due to item missingness

^Includes Hispanic/ Latino/ Latinx, Black or African American, Asian, Native Hawaiian or Pacific Islander, American Indian or Alaska Native

#### Total sample

In bivariate analyses among the total sample, older age at diagnosis, experiencing a relapse (versus did not relapse), and having an ostomy (versus no ostomy) were significantly associated with having a sexual health discussion. Females (versus males), white race/ethnicity (versus survivor of color), and lower level of education (high school graduate or less versus some college or more) were significantly negatively associated with having a sexual health discussion. In multivariable analysis among the total sample, age of diagnosis and experiencing a relapse retained their significance in the adjusted model. Female sex was negatively associated with reporting a sexual health discussion among the total sample.

#### Females

In bivariate analyses among females, older age of diagnosis and having an ostomy were significantly associated with having a sexual health discussion. White race/ethnicity (versus survivor of color) was significantly inversely associated with having a sexual health discussion. In multivariable analysis, older age of diagnosis was significantly associated with sexual health discussion.

#### Males

In bivariate analyses among males, older age at diagnosis, colon cancer (versus rectal cancer), experiencing a relapse (versus did not relapse), and having an ostomy (versus no ostomy) were significantly associated with having a sexual health discussion. In multivariable analysis, older age at diagnosis and experiencing a relapse retained their significance in the adjusted model.

## Discussion

The prevalence of both FSD and ED among early-onset CRC survivors was high, with 8 in 10 females reporting FSD and almost all males reporting ED, yet only 5 in 10 females and 6 in 10 males reported a sexual health discussion with their provider. These rates suggest that despite existing guidelines, a substantial portion of early-onset CRC patients are not receiving counseling on potential treatment-related late effects that could impair their sexual health.

It is notable that despite the prevalence of FSD and ED in this sample, sexual health discussions occurred for only half of surveyed patients. Similarly, in a study that included adult-onset CRC survivors (mean 58.5 years), only 41% reported that their provider informed them of potential impacts of cancer on their sexual function [13]. Patient knowledge gaps surrounding sexual function are common [29], and patient-level barriers to discussing sexual health suggest if discussions are not initiated by providers, they are unlikely to occur [14, 16, 17]. While the present study did not measure patient satisfaction with sexual health discussions, when these discussions do occur, over 70% of CRC survivors are satisfied with the information received [13]. In prior research, prostate cancer survivors report a preference to receive information on late effects from their oncologist and information on sexual health from their primary care provider, oncologist, or through written or online resources [30]. When informed of potential impacts to sexual health, patients may prioritize sexual rehabilitation by utilizing simple, yet effective strategies (i.e., moisturizers, dilators, pelvic floor physical therapy) to improve their sexual functioning after treatment [31, 32]. As such, guideline-concordant [11, 12] sexual health discussions have potential to improve patient outcomes, yet in the present study, their prevalence was suboptimal.

Over 80% of female early-onset CRC survivors reported sexual dysfunction in the present study, which is consistent with that of prior research among adult-onset female rectal cancer survivors (81%; mean 57.8 years) [33]. The present study utilized the FSFI-6, which has the advantage of brevity, allowing for rapid and efficient screening for FSD. However, despite its demonstrated reliability [26], this measure has not been used among cancer survivors. Therefore, direct, cancer-specific FSFI-6 comparisons are difficult to make from this work. Moving forward, future use of the FSFI-6 can provide context for patient scores identified in practice.

One of the most striking findings was the high prevalence of ED, with nearly every male reporting sexual dysfunction in the present study. The mean IIEF-5 score (13.6; representing mild to moderate ED [12 to 16]) [27] is consistent with prior research among male rectal cancer survivors, however, the overall prevalence of ED (94.5%) appears higher in the present study than in previous research. For example, in one study among early-onset male rectal cancer survivors (mean 44 years), patients had a mean IIEF-5 score of 14.7, similar to the present study [34]. In this same study, 46.2% reported mild (7.7%), moderate (2.6%), or complete (35.9%) ED, however, the IIEF-5 cutoff scores used differed from those in the present study and likely underestimate the prevalence of ED in their sample [27, 34].

In the present study, older age at diagnosis was significantly associated with a higher likelihood of reporting a sexual health discussion among both male and female survivors. As such, an age disparity in sexual health discussion may exist among patients on the younger end of the early-onset age range compared to patients on the older end of the early-onset age range. While older patients may feel more comfortable bringing up sexual health to their providers than younger patients, early-onset patients are uniquely prone to sexual dysfunction due to various factors such as negative change in body image, feelings of attractiveness, and sexual and romantic adversities [21, 35]. Written or online resources may be particularly beneficial for cancer patients who experience discomfort initiating or engaging in sexual health discussion [14, 30]. Further, digital health interventions among young cancer patients are gaining traction and may be beneficial for young patients who are often highly digitally connected [36, 37]. Overall, these findings indicate a need to prioritize sexual health inquiries with younger-aged patients who may not actively vocalize their concerns.

Overall, female early-onset CRC survivors in the present study were roughly 50% less likely to report a sexual health discussion than males. This may, in part, be due to stigma associated with female sexuality in which females feel discomfort discussing their sexual health [38, 39]. Despite the low prevalence of sexual health discussions with females, in past studies, female survivors have reported more sexual problems than male survivors [40], indicating a vast need for sexual health counseling among females. As such, the present findings and those of prior research [19, 20, 38–40] suggest female survivors are likely to be disproportionately affected by a lack of sexual health counseling and require targeted interventions to increase uptake of sexual health care.

Males who experienced a relapse were significantly more likely to report a sexual health discussion than males who did not experience a relapse. This may be due to the increased likelihood of treatment in relapse that can lead to sexual impairment (i.e., chemotherapy or radiation [41]), as well as increased healthcare utilization compared to non-relapsed patients, representing both a greater need and more opportunity for sexual health discussions [42]. While males who experienced a relapse appear more likely to report a sexual health discussion, ASCO guidelines recommend that sexual health discussions occur at time of diagnosis as timely intervention is likely to enact most positive outcomes [11, 12]. As such, timely sexual health discussion is needed to impart actionable outcomes for young patients.

Interestingly, the presence of an ostomy, a common comorbidity among CRC survivors with potential negative physical and psychological impacts on sexual health (e.g., body image, relationships with partners), was significantly associated with having a sexual health discussion among both males and females [43, 44]. While this finding suggests ostomy patients are more likely to receive support through sexual health discussion, overall discussion rates remain low, and it is unclear whether those discussions were anticipatory or reactive and who initiated them. As CRC survivors may experience survivorship challenges (e.g., adjusting to ostomy use) unique to their cancer experience, the effectiveness of sexual health counseling tailored to patient medical outcomes rather than the delivery of general sexual health information warrants exploration.

The present study has several strengths and some limitations. These findings contribute to the limited existing literature on sexual health and discussions surrounding early-onset CRC survivors. Data were acquired through a national young adult CRC advocacy organization, likely capturing survivors from different regions of the United States representing a variety of healthcare systems. However, this study may also represent a possible sampling bias toward patients who were digitally connected, familiar with the internet, and English-speaking. The extremely high prevalence of sexual dysfunction did not present sufficient variability for regression modeling to identify correlates of dysfunction among early-onset CRC survivors. Future research should also examine the content of sexual health discussions among those that do occur to characterize their level of guideline-concordance to facilitate optimal outcomes.

## Conclusion

Sexual dysfunction among early-onset CRC survivors is high and reported rates of sexual health discussion are suboptimal to address this late effect of cancer. The high prevalence of sexual dysfunction found in this study suggests that screening, access to resources, and timely intervention may be inadequately provided to early-onset CRC survivors. Providers who deliver individually tailored health care, normalize sexual issues and discussions, and have an effective referral system can facilitate optimal sexual health care for early-onset CRC survivors [10]. While many aspects of sexual dysfunction are non-specific and are shared by survivors of diverse cancer types, others are highly characteristic of the CRC population such as having an ostomy or experiencing stool incontinence [43–46]. With potential for adversely affecting sexual health both physically and psychologically, these require particular attention by the treating CRC provider [43, 44]. Overall, the findings reported here can help target patients at risk for not receiving sexual health counseling and can inform intervention development to increase provider awareness for preventing and managing sexual health challenges among early-onset CRC survivors.

## Data Availability

Please contact the corresponding author for information on the data set.
